# The Reciprocal Effect of Psychosocial Aspects on Nurses' Working Conditions

**DOI:** 10.3389/fpsyg.2017.01386

**Published:** 2017-08-15

**Authors:** Krystyna Kowalczuk, Elżbieta Krajewska-Kułak, Marek Sobolewski

**Affiliations:** ^1^Department of Integrated Medical Care, Medical University of Bialystok Bialystok, Poland; ^2^Faculty of Management, Rzeszow University of Technology Rzeszow, Poland

**Keywords:** psychosocial conditions, nurse, demands, well-being, stressors

## Abstract

**Objectives:** Psychosocial work risks are most often considered in the context of occupational stress. The aim of this article is to evaluate the correlations between different aspects of nurses' psychosocial working conditions.

**Materials and Methods:** The study was conducted using the questionnaire: Psychosocial aspects of work. A total of 789 nurses working in inpatient health care facilities in Bialystok were included in the study. Correlation analysis was performed by determining Spearman's correlation coefficient.

**Results:** Correlations between the primary scales, such as job demands, control, social support, well-being, and expectations of changes, were evaluated. The weakest correlation was shown between the assessment of job demands and other work aspects. The strongest correlation was found between the ability to control and social support. Perception of the need for changes was influenced by the assessment of job demands, components of the control scale and, most of all, the scale of social support. A strong correlation was found between physical and psychological well-being and support from superiors and coworkers.

**Conclusions:**

1. The state of well-being had no effects on nurses' assessment of the demands they were faced with. Nurses' well-being depended only on social support provided by their superiors and colleagues, the sense of being able to have an effect on the performed work, minimal conflicts, and absence of overload.
2. Management should enable adequate working conditions in order to ensure nurses' physical and psychological well-being, as both these aspects were closely correlated.
3. Poor social support, lack of a sense of control over one's work, conflicts, and work overload were factors that promoted nurses' expectations of changes.

## Introduction

The main aim of the research described in the article is to evaluate the correlations between different aspects of nurses' psychosocial working conditions to investigate the factors that affect the well-being of nurses in the workplace. Further on to examine which psychosocial factors cause nurses to expect changes in their workplace. And finally, what actions should be taken by management to ensure that the nurses have decent physical and mental conditions in their workplace.

Many researchers point out that nurses experience a great deal of stress during work because of excessive workload, role ambiguity, and interpersonal conflict. This affects negatively their mental and physical health, and can therefore lead to burnout and indirectly hamper the patient's safety (Pisanti et al., 2011, 2015, 2016; Rudman et al., 2012; Panagopoulou et al., 2015; Welp et al., 2015; Giorgi et al., 2016).

So far in Poland no standards have been set for the employment of nurses in hospitals. The financial difficulties of individual institutions and the overall decline in the number of professionally active nurses mean that the number of nurses employed per 1,000 inhabitants is 5.2 in Poland and is significantly lower than in other European countries, for example in Sweden it is 10.2 in Germany 13 and in Norway 18, 8 (Healthcare Personnel Statistics - Nursing and Caring Professionals, 2016). This is the main reason why nurses in Poland are more burdened at work than their colleagues in other European countries (Basinska and Wilczek-Rużyczka, 2011; Borowiak et al., 2011; Dåderman and Basinska, 2016).

The International Labor Organization (ILO) defines psychosocial risks as the interaction between work content, organization and management of the work process, taking into account the competences and individual needs of a person (European Agency for Safety and Health at Work Topic Centre Risk Observatory, 2007). Psychosocial work risks are most often considered in the context of occupational stress. A number of studies on the division and classification of the above mentioned risks may be found in the literature. Classification is usually based on stress models or empirical data containing lists of known potential stressors. The emerging risks associated with new forms of work, e.g., telework, temp work have not yet been covered by studies (Hasselhorn et al., 2008; Widerszal-Bazyl, 2009; Leka et al., 2010; Potocka, 2010; Parent-Thirion et al., 2012; Hegney et al., 2015; Cox et al., 2016).

There are many divisions of psychosocial risks. One of the more useful seems to be the division proposed by Cox and Cox in 1993 and modified by Hasselhorn et al. (2008). It presents the division of psychosocial risks into several groups, taking into account the work content, work pace and overload, work schedule, control, environment and equipment, the organizational culture, role in the company, the course of a career, and the work-home relationship (Hasselhorn et al., 2008; Widerszal-Bazyl, 2009).

The creators of the concept of sustainable work design based on the theory of the organization of workplace, stress, and ergonomic principles have identified several factors that may potentially lead to stress load. According to them, various organizational factors, such as demands, overload, underload, control, ergonomic mismatch, or physical factors may not be the only potential stressors. They also noticed that stressors can result from individual human characteristics: personality, health status, setting inadequate goals, and acquired experiences (Basinska and Wilczek-Rużyczka, 2011; Borowiak et al., 2011; Dåderman and Basinska, 2016). According to this theory, the imbalance between the above mentioned factors and the individual characteristics of an employee can lead to functional changes at the psychological, physical, and behavioral level and, consequently, to stress (Cox and Cox, 1993; Leka et al., 2010; Potocka, 2010; Hegney et al., 2015).

For at least 20 years, the psychosocial work environment has been analyzed in the literature in terms of its effects on human health using Karasek's demands-control model (Karasek and Theorell, 1990). The model considers demands resulting from the performed work, taking into account the pace of work, conflict of the position, and job control, distinguishing cognitive and behavioral control. It is assumed in this model that high levels of control and low-to-medium levels of demands are perceived in a positive manner by employees and do not pose a threat to health. High levels of demands and low levels of control, on the other hand, may reduce the functioning of an employee in the working environment and entail negative health-related consequences.

Literature data indicate that full-blown stress is preceded by behavioral, psychological, and medical problems (Cieślak and Widerszal-Bazyl, 2000; Hasselhorn et al., 2008; Widerszal-Bazyl, 2009; Basinska and Wilczek-Rużyczka, 2011; Borowiak et al., 2011; Cox et al., 2016; Dåderman and Basinska, 2016). Behavioral problems, such as alcohol and/or drug abuse, smoking tobacco, aggressive behavior, which are most noticeable to the environment, are first to occur. Psychological consequences are related to family life, sexual, and sleep disorders. Finally, medical issues lead to more rapid onset of diseases.

## Materials and methods

### Sample

Women accounted for the vast majority of respondents (721, i.e., 90%). It can be assumed that the study population was homogenous in terms of sex. Individuals with higher education, who accounted for nearly 45% of all respondents, dominated in the study group. Approximately one in ten respondents held a managerial position. Nurses' ages ranged from 20 to 58 years, with mean age of 41 years and deviation of ±9 years. Every fourth respondent was over 48 years old. Average seniority was about 17 years, whereas average seniority at current position was shorter than total seniority by about 2 years. The nurse professional group is strongly feminized (not only in Poland) and it is precisely this group structure, with the apparent dominance of women, that can be the basis for correct conclusions (Borowiak et al., 2011).

### Procedure

The study was conducted from June 2012 to March 2013 in Bialystok. It included 789 nurses working in inpatient health care facilities. As some of the respondents did not reply to all questions in the questionnaire, the number of analyzed cases in some of the summary lists may be a few to several dozen less than the overall population. Participation was voluntary, and all procedures were approved by the Local Bioethics Committee of the Medical University of Bialystok.

### Instrument

Respondents were asked to complete a standardized questionnaire: “Psychosocial aspects of work” (Cieślak and Widerszal-Bazyl, 2000; Potocka, 2012) which included 118 questions and comprised of 6 parts:

Part I. Scale of Demands (D). What does your work demand?—25 questions.
Part II. Scale of Control (C). To what extend can you influence what happens at work?—20 questions.
Part III. Scale of Social Support (SS). What support can you expect?—16 questions.
Part IV. Scale of Well-being (W). How is your well-being?—22 questions.
Part V. Scale of Desired Changes (DC). Do you expect any changes at work?—20 questions.
Part VI. Metrical data. Who are you and what is your company?—15 questions.

Using the calculation methods described in the test key manual (Cieślak and Widerszal-Bazyl, 2000) the following scales are taken: Scale of Demands (D), Scale of Control (C), Scale of Social Support (SS), Scale of Well-being (W), Scale of Desired Changes (DC). These scales are numerical (continuous variable) and they take values from the range 1 to 5 points. For the purposes of our research, the results were calculated for each scale and subscale in accordance with the coding principle and key provided by Cieślak et al. Next, the scores for answers to the questions included in a given scale were summed. The resulting totals were divided by the number of questions to which the respondent answered to receive average scores for a scale or subscale. Thus obtained values were compared against standards. The higher the score after summing the scores, the higher the intensity of a given item—demands, control, social support, well-being, and desired changes. The questionnaire is in accordance with standards for eight professional groups, including nurses. Scale reliability, measured using Cronbach's alpha index, ranged between 0.82 and 0.94, and therefore is considered satisfactory.

### Analysis

The choice of statistical methods depended on the nature of the evaluated characteristics. The numerical nature of the scales based on the questionnaire determines the choice of statistical methods (descriptive statistics, correlation analysis, regression model). For the evaluation of the effects of a nominal characteristic on working condition assessment, statistical analysis involved a comparison of mean values in the chosen groups as well as an assessment of statistical significance of differences between the groups using an appropriate test. Due to the fact that distribution of working conditions ratings in each category was comparable to normal distribution, and there were no significant distribution asymmetries or deviations, the analysis of variance test was used. For the evaluation of the effects of numerical characteristics on working conditions, correlation analysis determining Spearman's correlation coefficient was used.

An attempt was made to assess the impact of selected independent factors on the assessment of psychosocial working conditions. Based on the research conducted, the following factors were selected that could influence the values of psychosocial working conditions measures: type of hospital ward, age, education, occupied position (managerial or not). A general linear model (GLM) was used to evaluate the significance of the influence of individual factors on the psychosocial working conditions measures. The table gives the values of the estimated parameters together with the assessment of their statistical significance. The quality of fit in the form of determination coefficient (*R*^2^) is also given. It should be noted that attempts to include interactions of the second degree between the factors in the model shown that these interactions were not statistically significant and therefore were not included in the final version of the model.

## Results

The nurses' assessment of work demands was high (mean score of about 3.5). The ability to control one's own work and the level of social support were assessed at an average level (mean scores of 3.01 and 3.06, respectively). The rating of life satisfaction was rather high (mean score of 3.62). The assessment of the scale of desired changes, which should occur in their work, according to the respondents, was also high. This factor received a mean score of 3.57.

Table 1 shows data on all scales and subscales of the questionnaire of work condition assessment. Analyzing the results, it is worth noting significant differences between the components of some of the scales. For example, the level of psychophysical demands was highly rated (a score of nearly 4.3), while much lower scores were obtained by the level of intellectual demands, and the least scores were obtained by the factor of conflicts and overload at work due to excess responsibilities (mean score of only 2.71). As for other interesting findings, it is worth noting a higher rating of colleague support compared to superiors' support (a mean score of 3.19 vs. 2.83).

**Table 1:** Subjective assessment of work conditions based on components of individual scales.

**Assessment of work conditions**	***N***	***x***	**Me**	***S***	***c*_25_**	***c*_75_**	**Min**	**Max**
Scale of demands	789	3.49	3.50	0.39	3.24	3.76	1.40	4.56
Intellectual demands	789	3.29	3.22	0.54	2.89	3.67	1.11	4.89
Psychophysical demands	789	4.26	4.33	0.49	4.00	4.56	1.44	5.00
Conflictuality and overload	789	2.71	2.67	0.63	2.33	3.17	1.00	4.67
Scale of control	788	3.06	3.05	0.43	2.80	3.30	1.85	4.40
Behavioral control	788	2.45	2.40	0.54	2.10	2.80	1.20	4.40
Cognitive control	788	3.75	3.78	0.54	3.44	4.11	1.33	5.00
Scale of social support	784	3.01	3.00	0.70	2.63	3.44	1.00	5.00
Support from superiors	784	2.83	3.00	0.82	2.31	3.38	1.00	5.00
Support from colleagues	784	3.19	3.25	0.73	2.75	3.63	1.00	5.00
Scale of well-being	783	3.62	3.64	0.54	3.23	4.05	1.64	5.00
Physical well-being	783	3.71	3.73	0.63	3.27	4.18	1.64	5.00
Mental well-being	783	3.53	3.55	0.53	3.18	3.91	1.64	5.00
Scale of desired changes	779	3.57	3.60	0.63	3.15	4.05	1.00	4.90
Need for change	784	3.61	3.63	0.65	3.16	4.05	1.00	5.00

*(N), number of assessments; x̄, arithmetic mean—variable average; Me, median—half the measures have lower values and half higher from the median; Max, maximum value; min, minimum; S, standard deviation (s)—a measure of “average” deviation from the mean value; 25th and 75th percentile, first and third quartiles*.

**Figure 1:**
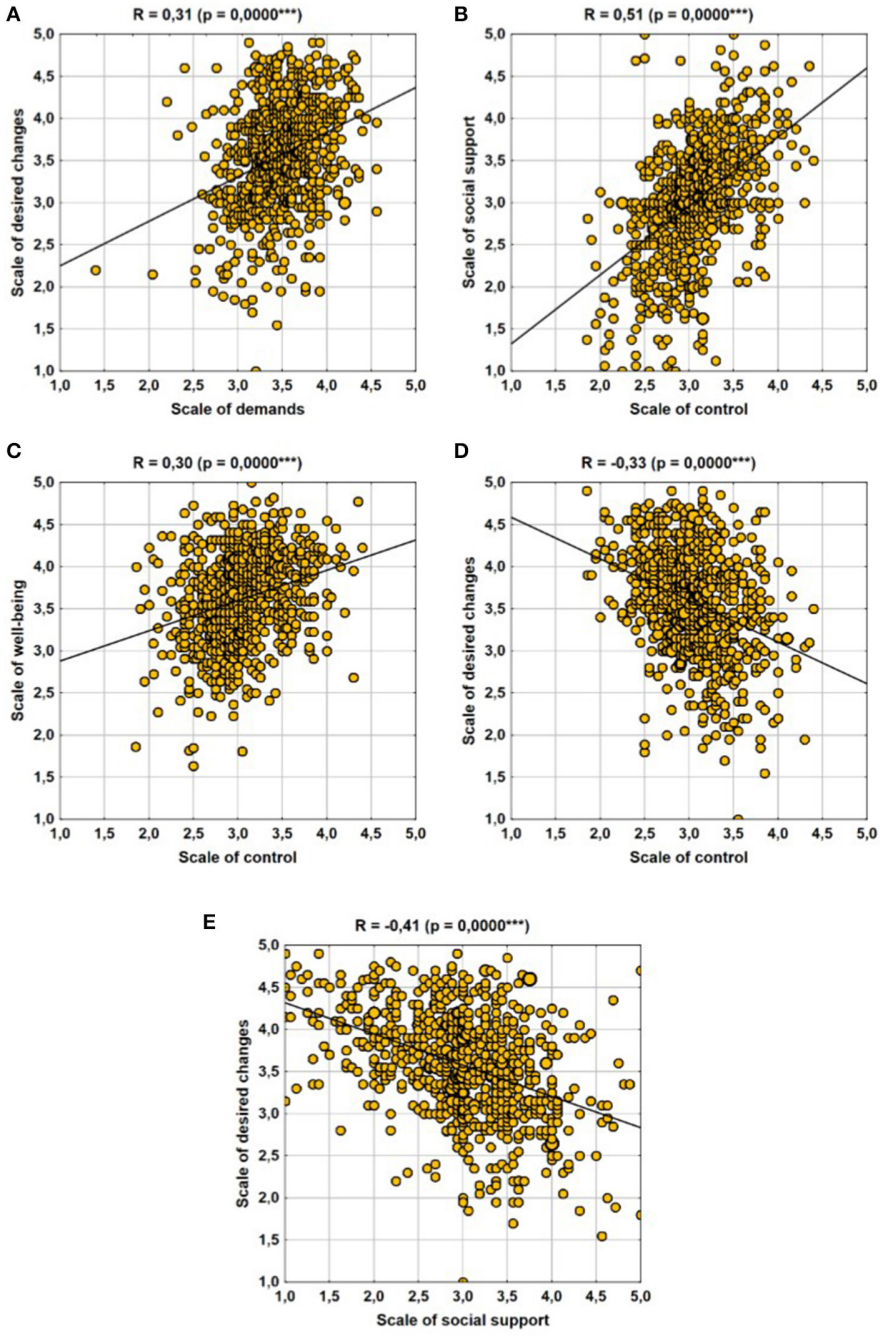
Strongest correlations between the main scales. **(A)** Scale of desired changes–Scale of demands, **(B)** Scale of social support–Scale of control, **(C)** Scale of well-being–Scale of control, **(D)** Scale of desired changes–Scale of control, and **(E)** Scale of desired changes–Scale of social support.

We analyzed the correlations between the ratings of different aspects of work conditions (Table 2). First, correlations between the main scales, i.e., demands, control, social support, well-being, and expectations of changes, were evaluated. The weakest correlation was shown between the assessment of job demands and other work aspects. It showed a very poor correlation with the assessment of social support and slightly stronger correlation with the assessment of the need for changes. Respondents who rated their work demands higher more often expected changes (leading to reduced demands?). The perception of the need for changes is also influenced by the assessment of control or rather modification of one's way of working—individuals who did not notice such opportunities wanted to change it (negative correlation coefficient ρ = −0.33). Individuals with low levels of social support also expected changes (ρ = −0.41). As for other correlations, it is worth mentioning that the strongest correlation was between the scale of control over one's own work and social support. Correlation between these two positive aspects of work were relatively strong (ρ = 0.51). The strongest correlations are presented in the form of scatter plots (Figure 1).

**Table 2:** Correlations between the main scales.

**Correlation**	**Scale of demands**	**Scale of control**	**Scale of social support**	**Scale of well-being**	**Scale of desired changes**
Scale of demands	1	−0.01 (*p* = 0.8879)	−0.15 (*p* = 0.0000^***^)	−0.06 (*p* = 0.0785)	0.31 (*p* = 0.0000^***^)
Scale of control	−0.01 (*p* = 0.8879)	1	0.51 (*p* = 0.0000^***^)	0.30 (*p* = 0.0000^***^)	−0.33 (*p* = 0.0000^***^)
Scale of social support	−0.15 (*p* = 0.0000^***^)	0.51 (*p* = 0.0000^***^)	1	0.22 (*p* = 0.0000^***^)	−0.41 (*p* = 0.0000^***^)
Scale of well-being	−0.06 (*p* = 0.0785)	0.30 (*p* = 0.0000^***^)	0.22 (*p* = 0.0000^***^)	1	−0.16 (*p* = 0.0000^***^)
Scale of desired changes	0.31 (*p* = 0.0000^***^)	−0.33 (*p* = 0.0000^***^)	−0.41 (*p* = 0.0000^***^)	−0.16 (*p* = 0.0000^***^)	1

*** *correlation statistically very higly significant*.

This was followed by an analysis of factors influencing respondents' well-being (Table 3). The majority of work aspects were related to the assessment of well-being, although these correlations were rather weak (sporadically |*R*| > 0.40). Among the aspects of working conditions that affected the level of well-being (in general as well as when divided into psychological and physical components), the following should be mentioned in particular:

- the negative impact of conflicts and overload (*R* = −0.31 for total well-being);
- the positive impact of the ability to influence one's own work (scale of control), with particularly significant effects of cognitive control (strongest correlation *R* = 0.37 for psychological well-being);
- the positive impact of social support, especially on psychological well-being (it should be noted that the strength of the impact was similar for both colleague and superior support);
- the scale of desired changes was of minor importance, in general, those who expected changes showed slightly lower well-being.

**Table 3:** Subjective assessment of respondent' well-being.

**Other elements of work conditions**	**Assessment of well-being**		
	**Total**	**Physical well-being**	**Mental well-being**
Scale of demands	−0.06 (**p** = 0.0785)	−0.07 (**p** = 0.0478^*^)	−0.04 (**p** = 0.2697)
Intellectual demands	−0.01 (**p** = 0.7429)	−0.04 (**p** = 0.3063)	0.02 (**p** = 0.5998)
Psychophysical demands	0.05 (**p** = 0.1732)	0.04 (**p** = 0.2565)	0.06 (**p** = 0.0924)
Conflictuality and overload	−0.31 (**p** = 0.0000^***^)	−0.28 (**p** = 0.0000^***^)	−0.31 (**p** = 0.0000^***^)
Scale of control	0.30 (**p** = 0.0000^***^)	0.24 (**p** = 0.0000^***^)	0.33 (**p** = 0.0000^***^)
Behavioral control	0.10 (**p** = 0.0050^**^)	0.06 (**p** = 0.1152)	0.13 (**p** = 0.0002^***^)
Cognitive control	0.34 (**p** = 0.0000^***^)	0.28 (**p** = 0.0000^***^)	0.37 (**p** = 0.0000^***^)
Scale of social support	0.22 (**p** = 0.0000^***^)	0.15 (**p** = 0.0000^***^)	0.26 (**p** = 0.0000^***^)
Support from superiors	0.21 (**p** = 0.0000^***^)	0.16 (**p** = 0.0000^***^)	0.24 (**p** = 0.0000^***^)
Support from colleagues	0.20 (**p** = 0.0000^***^)	0.14 (**p** = 0.0001^***^)	0.25 (**p** = 0.0000^***^)
Scale of desired changes	−0.16 (**p** = 0.0000^***^)	−0.14 (**p** = 0.0001^***^)	−0.14 (**p** = 0.0001^***^)
Need for change	−0.13 (**p** = 0.0002^***^)	−0.12 (**p** = 0.0006^***^)	−0.12 (**p** = 0.0007^***^)

* correlation statistically significant;

** correlation statistically very significant;

*** *correlation statistically very higly significant*.

Thirdly, we investigated factors that influenced expectations of change (Table 4). The perception of the need for work-related changes was affected by both the assessment of demands as well as the components of the control scale and the social support scale. The greatest impact (yet still relatively weak) was shown for support. Individuals with low social support expressed a greater need for changes. Support from superiors was particularly important (*R* = −0.44). Similarly, those who perceived the opportunity to influence and control their own work did not want changes in their work (*R* approx. −0.30). A high level of demands (conflicts and overload in particular *R* = 0.36) was a factor that promoted the increase of expectations related to work changes.

**Table 4:** Effect of factors on the need for changes.

**Assessment of demands, control and support**	**Assessment of desired changes**	
	**Scale of the desired changes**	**Need for change**
Scale of demands	0.31 (*p* = 0.0000^***^)	0.31 (*p* = 0.0000^***^)
Intellectual demands	0.14 (*p* = 0.0001^***^)	0.14 (*p* = 0.0001^***^)
Psychophysical demands	0.18 (*p* = 0.0000^***^)	0.19 (*p* = 0.0000^***^)
Conflictuality and overload	0.36 (*p* = 0.0000^***^)	0.35 (*p* = 0.0000^***^)
Scale of control	−0.33 (*p* = 0.0000^***^)	−0.33 (*p* = 0.0000^***^)
behavioral control	−0.25 (*p* = 0.0000^***^)	−0.25 (*p* = 0.0000^***^)
Cognitive control	−0.27 (*p* = 0.0000^***^)	−0.26 (*p* = 0.0000^***^)
Scale of social support	−0.41 (*p* = 0.0000^***^)	−0.41 (*p* = 0.0000^***^)
Support from superiors	−0.44 (*p* = 0.0000^***^)	−0.45 (*p* = 0.0000^***^)
Support from colleagues	−0.28 (*p* = 0.0000^***^)	−0.27 (*p* = 0.0000^***^)

*** *correlation statistically very highly significant*.

To investigate which nurses expect changes in work and to explain the mechanism of this phenomenon regression analysis was applied. To this end, the independent factors (age, type of hospital ward, education, and position) influence on expected changes in work was examined (Table 5). The only statistically significant factor turned out was “age”—with increasing awareness of the need for changes in the way a nurse operates at work. When comparing two groups with age differences by 10 years, it is expected to be about 0.06 points higher on the need for changes in the older age group.

**Table 5:** Impact of independent factors on scale of desired changes.

Regression model for the scale of desired changes *R*^2^ = 2.2% *F* = 2.36, *p* = 0.0215^*^			
**Independent factor**	**Rated effect**	**B**	***p***
Age	1 year	0.006	0.0268*
Hospital ward	Operating block vs. internal	−0.042	0.4581
	Outpatient care vs. internal	0.002	0.9732
	Surgical vs. internal	−0.050	0.2113
Education	Higher vs. secondary	−0.017	0.6358
	Postsecondary vs. secondary	0.017	0.6311
Managerial position	No vs. yes	0.005	0.8920

*B, regression coefficient; p, result of statistical significance test*.

Continuation of analysis of expectations of changes in work was an attempt to describe the perception of the need of changes in nurses' work in the category of socio-occupational factors and other measures of psychosocial aspects of work. Only the age was introduced into the regression model (based on analysis shown in Table 5) and the other four measures of psychosocial aspects of work. It turned out that knowing the values of: the scale of demands, the scale of control and the scale of social-support the influence of age and scale of well-being become non-significant on the magnitude of the desired change scale (Table 6).

**Table 6:** Impact of independent factors and other measures of working conditions on expectations regarding changes in work.

Regression model for the scale of desired change *R*^2^ = 27.2% *F* = 54.9, *p* = 0.0000^***^			
**Independent factor**	**Rated effect**	***B* (β)**	***p***
Age	1 year	0.002 (0.03)	0.2945
Scale of demands	1 point	0.457 (0.28)	0.0000^***^
Scale of control	1 point	−0.285 (−0.19)	0.0000^***^
Scale of social support	1 point	−0.246 (−0.27)	0.0000^***^
Scale of well-being	1 point	−0.010 (−0.01)	0.7914

*B, regression coefficient; β, standardized regression coefficient; p, result significance test*.

*** *correlation statistically very highly significant*.

**Figure 2:**
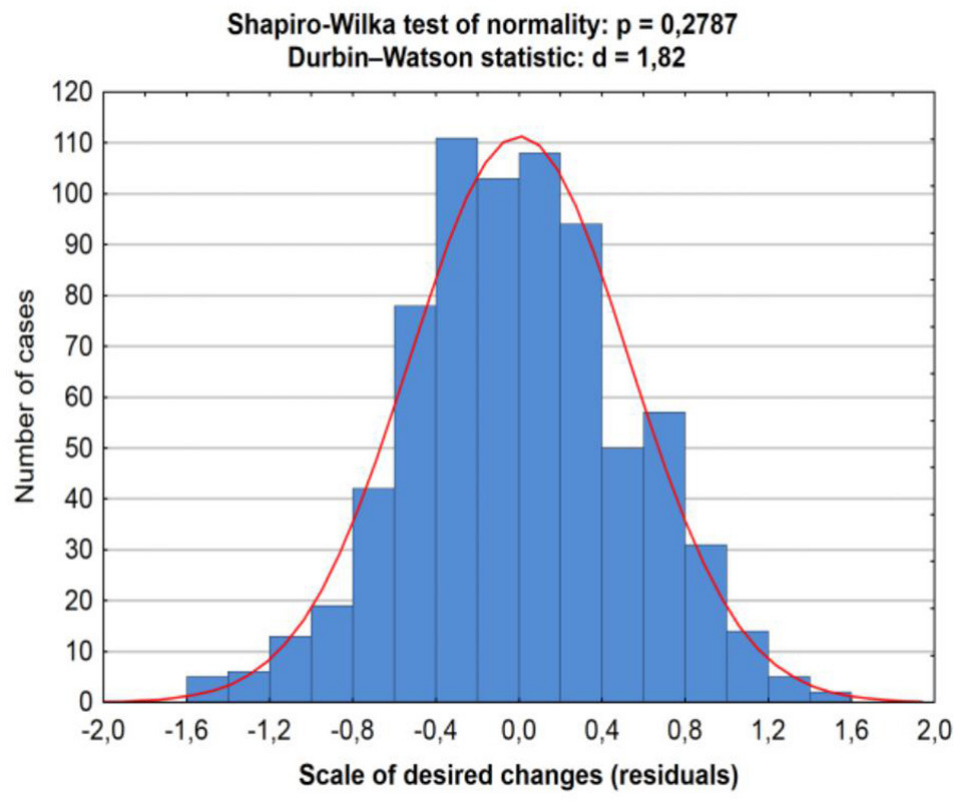
Shapiro-Wilk and Durbin-Watson tests for Regression model for the scale of desired change.

An analysis of residuals in the regression model was performed in terms of their normality, randomness and other desirable properties. The results were satisfactory, which means that the results of the regression analysis should not be falsified. An example of a graph showing the residual distribution for the model described in Table 6 is shown on Figure 2. It presents the Shapiro-Wilk normality test and the Durbin-Watson statistic test, which indicate the desirable residual distribution (normality and lack of autocorrelation).

Among the factors considered, the most significant is the scale of demands—with an increase in the scale of demands by 1 point, the assessment of the necessity of changes in the work of nurses is also increasing - on average about 0.457 points. On the other hand, with the increase in the assessment of the ability to control (in a positive sense) of their work and the assessment of social support in work, the perception of the need for changes in the work of the nurse falls. A 1 point increase in scale of control translates into a 0.285 (average) decrease on scale of change, while a 1 point increase in the scale of social support is an average decrease of scale of change of about 0.246 points. The assessment of the influence of individual independent variables on the scale of change can also be carried out on the basis of standardized regression coefficients (β). It turns out that the magnitude of the impact of the scale of demands and the scale of social support on the scale of desired changes is similar, and influence of the scale of control is less important.

**Figure 3:**
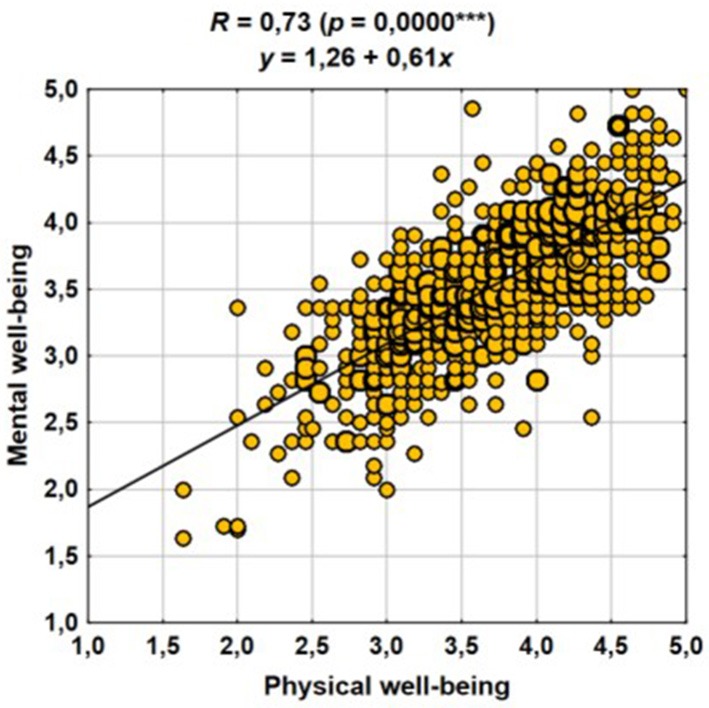
Correlation of psychological and physical well-being.

Next, we investigated the relationship between physical and psychological well-being. Both these components of well-being were relatively closely correlated with each other. Naturally, those at the same level of physical well-being showed a relatively broad spectrum of psychological well-being ratings (and vice versa); however, it can be for example stated with a relatively high certainty (Figure 3) that those who rated their physical well-being at 4.5–5.0 points would also rate their psychological well-being higher compared with those who rated their physical well-being at 2.5 or 3.0 points.

The header of the graph (Figure 3) contains the regression equation, which indicates that the increase in the rating of physical well-being by 1 point resulted in an increased rating of psychological well-being by ~0.6 points.

The last identified correlation was found between superiors' and colleagues' support. A large majority of respondents rated superiors' support lower compared with colleagues' support. A regression equation was formulated, and it indicated that an increase in the rating of superiors' support by 1 point translated into an increase in the rating of colleagues' support by an average of approximately 0.6 points.

## Discussion

In this study, we analyzed the relationships between the ratings of different aspects of working conditions. It seems interesting whether a high rating of demands affects the level of well-being (there are grounds to believe that this effect may be negative). Similarly, it can be expected that a high rating of the level of social support may translate into psychological well-being. Finally, it is interesting who expected changes at work: those who were pleased with themselves or perhaps it depends on the assessment of the level of demands (and if this is the case, were changes expected by those who considered the level of demands as too high or too low?). We also investigated how different components of the same scales correlated with one another; for example, if there was a correlation between physical and psychological well-being, whether the assessment of coworker support was strongly correlated to superiors' support.

The concept of control is presented in the literature from three perspectives. Control can be perceived as a feature of the job, which means that one has an effect on one's own work, which in turn enables reducing the negative effects of stress. Perceiving control as a personality trait means a way an individual assesses the situation at work based on acquired experience. In the third perspective, control exists as a psychological phenomenon, a conviction of an individual that it is possible to choose a purpose as well as the method to achieve it (Head et al., 2006; Davey et al., 2009; Adriaenssens et al., 2011; Potocka, 2012; Ferri et al., 2016). A sense of poor control or lack of control over the performed work creates stress conditions related to one's own actions in situations of threat to other's life and health. Additionally, the lack of a nurse's specific role in the professional hierarchy is a source of chaos in everyday work and causes feelings of insecurity. Tartas et al. (2009) showed that situations associated with a lack of control over the performed work were common in the work of nurses.

It seems interesting why nurses have little control over their work despite the fact that this is an independent profession. Studies conducted among Belgian nurses showed that the low levels of control over the performed work resulted from inappropriate management procedures in certain nursing situations (Adriaenssens et al., 2011). Studies, conducted by Roe (2008), Schaufeli et al. (2009), and Tang (2014) showed that high demands of employees with poor decision-making abilities and low level of social support were a source of high stress levels. A study conducted among Canadian nurses (Gelsema et al., 2016) found that high mental stress was associated with stress in a short time. It can be stated that employees have increased motivation for action and feel personal satisfaction if they have high control combined with high requirements and support (Karasek and Theorell, 1990). Studies conducted in Malaysia confirmed that the ability to control one's own work reduced the psychophysical demands, thus increasing well-being (Amin et al., 2014).

In our study, work-related control was analyzed as a feature of work that can be affected by the employee. Respondents assessed their influence on performing their work (control) as “average” (mean score of 3.06 on 1–5 scale). The performed analysis showed a strong correlation between feeling control over one's own work and nurses' psychological and physical well-being as well as support from superiors and coworkers, which is in line with the findings of the Malaysian studies (Amin et al., 2014). The strong need for changes at work corresponds with the results of the study conducted among Belgian nurses.

In our studies, social support was presented as the concept of perceiving “from where and whom one can receive assistance in critical situations.” We found that the level of social support received by the respondents from their superiors and colleagues had a positive impact on their psychological well-being. Similar results were obtained in Belgium, where the respondents were shown to receive significant support from their colleagues and less support from their superiors. According to the surveyed nurses, adequate support from their superiors would significantly increase their satisfaction and commitment to work, and thus contribute to stress reduction and increased well-being perception (Adriaenssens et al., 2011). Similarly, Tartas et al. (2009) showed that the support nurses received from their superiors and coworkers was poor, which resulted in high stress levels.

In our study, we have shown that the nurses who expected changes perceived the demands imposed on them as high. The study conducted in Belgium showed that the nurses were overloaded with quantitative and qualitative demands as well as time pressure, which was an additional predictor of physical fatigue. The surveyed nurses expected work improvements (Adriaenssens et al., 2011).

A cross-sectional study conducted in the Netherlands showed a mutual correlation between the emotional demands at work and well-being. Decreased psychological well-being may be compensated with increased fulfillment of the employee's emotional needs of, as well as increased perception of well-being and control over work. According to the authors, an increase in an employee's resources would have a positive effect on the perception of work demands and would increase well-being (De Jonge et al., 2008). In our study, we found no such correlations.

Studies conducted in Sweden showed that working with patients is associated with significant emotional exhaustion. High work overload had negative effects on the emotional and physical well-being of respondents (Sundin et al., 2007). Similar results were obtained in our studies, where we found that conflicts and overload had a negative impact on physical and psychological well-being.

Studies conducted in Klang Valley (Malaysia) among public hospital nurses showed that psychosocial factors had significant effects on the subjective feeling of somatic symptoms. Only in case of emotional job demands statistical analysis has not confirmed their influence on the occurrence of the physical symptoms. Furthermore, it was found that poor support from superiors and colleagues reduced well-being (Amin et al., 2014). This is highly consistent with our findings.

Freimann et al. (2016) showed, on the other hand, that there was no relationship between having control at nursing work and the scale of psychophysical overload. It was also found that the low levels of social support had no effects on the occurrence of health problems. While the first finding corresponds with our outcomes, the second is completely different if we consider “the occurrence of physical symptoms” as equivalent to “physical well-being” (Sundin et al., 2007; De Jonge et al., 2008; Dziąbek et al., 2013; Amin et al., 2014; Rotter et al., 2014; Adriaenssens et al., 2015).

Conflicts in the nursing environment are similar in many countries. This is due, among other things, to the inability of the nurses to manage inequalities from the managers of nurses, improper interpersonal relationships with doctors resulting from different perceptions of responsibilities and responsiveness (Higazee, 2015). Conflict situations also cause negative emotions caused by inadequate behavior of patients and nurses themselves (Wright et al., 2013). Conflicts are associated with one's own professional group, but they also result from inappropriate interpersonal communication and perception of physician incompetence. Nurses, due to their duties, are obliged to act according to a professional code, therefore they often suppress negative emotions resulting from the specificity of their work, e.g., multiple accidents, death of a patient, contact with the family. According to Dziąbek et al. (2013), coping with difficult and conflicting situations and the ability to express emotions promote physical and psychological well-being. Our findings confirmed the thesis of many other studies that conflicts have negative effects on well-being and demonstrated a strong correlation between physical and psychological well-being.

The outcomes of the subjective assessment of psychosocial risks leave no doubt that these factors negatively affect an employee. Therefore, it is advisable to introduce psychosocial risk monitoring in the workplace using standardized tools, as well as to inform employees of the outcomes of the risk research and assessment. In the long term, programs for stress prevention at the individual and company level should be developed and implemented.

The published results of statistical analyzes do not exhaust all possible hypotheses and can be greatly expanded. It was decided to focus on the selected aspects of the problem, which was conditioned by the selection of tools and the scope of statistical analyzes. Studies may be continued in other professional medical groups such as physicians, and then the results of the groups may be compared.

## Conclusions

1. The state of well-being had no effects on nurses' assessment of the demands they were faced with. Nurses' well-being depended only on social support provided by their superiors and colleagues, the sense of being able to have an effect on the performed work, minimal conflicts, and absence of overload.
2. Management should enable adequate working conditions in order to ensure nurses' physical and psychological well-being, as both these aspects were closely correlated.
3. Poor social support, lack of a sense of control over one's work, conflicts, and work overload were factors that promoted nurses' expectations of changes.

## Author contributions

KK—concept of the article, literature research, survey, data collection, results interpretation, drafting article, approval of the final version; EK—questionnaire approval, review of article drafts, results interpretation, approval of the final version; MS—questionnaire approval, statistical analysis, results interpretation, review of article drafts.

### Conflict of interest statement

The authors declare that the research was conducted in the absence of any commercial or financial relationships that could be construed as a potential conflict of interest.
